# HER2-Targeted Therapy—From Pathophysiology to Clinical Manifestation: A Narrative Review

**DOI:** 10.3390/jcdd10120489

**Published:** 2023-12-06

**Authors:** Svetoslava Elefterova Slavcheva, Atanas Angelov

**Affiliations:** 1 First Department of Internal Diseases, EC Cardiology, Faculty of Medicine, Medical University “Prof. Dr. Paraskev Stoyanov”, 9000 Varna, Bulgaria; atanas.a.atanasov@mu-varna.bg; 2 First Cardiology Clinic with Intensive Cardiology Activity, University Multiprofessional Hospital of Active Treatment “St. Marina”, 9000 Varna, Bulgaria

**Keywords:** cardiotoxicity, trastuzumab, pathophysiology, clinical manifestation

## Abstract

Trastuzumab is the primary treatment for all stages of HER2-overexpressing breast cancer in patients. Though discovered over 20 years ago, trastuzumab-induced cardiotoxicity (TIC) remains a research topic in cardio-oncology. This review explores the pathophysiological basis of TIC and its clinical manifestations. Their understanding is paramount for early detection and cardioprotective treatment. Trastuzumab renders cardiomyocytes susceptible by inhibiting the cardioprotective NRG-1/HER2/HER4 signaling pathway. The drug acts on HER2-receptor-expressing cardiomyocytes, endothelium, and cardiac progenitor cells (see the Graphical Abstract). The activation of immune cells, fibroblasts, inflammation, and neurohormonal systems all contribute to the evolution of TIC. A substantial amount of research demonstrates that trastuzumab induces overt and subclinical left ventricular (LV) systolic failure. Data suggest the development of right ventricular damage, LV diastolic dysfunction, and heart failure with preserved ejection fraction. Further research is needed to define a chronological sequence of cardiac impairments to guide the proper timing of cardioprotection implementation.

## 1. Introduction

Identifying human epidermal growth factor receptor 2 (HER2), elucidating its intracellular signaling mechanisms in tumor cells, and the advent of the recombinant anti-HER2 monoclonal antibody trastuzumab led to a transformative change in oncology research. In 2019, the Lasker Prize, called the American Nobel Prize, was awarded to three scientists: H. Michael Shepherd, Dennis J. Slamon, and Axel Ulrich. They were acknowledged for their groundbreaking accomplishments in inventing the first monoclonal antibody, trastuzumab, which targets a tumor-associated protein, and in creating a life-saving therapy for breast cancer [1]. The discovery had a significant impact on the approach to targeting tumor cells. The development of this treatment modality provided the opportunity to change prognostic outcomes in HER2-overexpressing breast cancer patients, 85% of whom received a predicted 10-year survival [2]. Trastuzumab has become a standard medication for the treatment of HER2-positive early-stage and metastatic breast cancer [3,4,5]. Trastuzumab has shown efficacy in approximately 20% of individuals diagnosed with gastric cancer.

Slamon et al. (2001) conducted a landmark study on HER2-positive metastatic breast cancer and reported on the adverse cardiac effects of trastuzumab [6]. These findings were unexpected given that the medicine exclusively targeted tumor cells [6]. The study revealed that 27% of patients who underwent treatment using a combination of trastuzumab, anthracyclines, and cyclophosphamide exhibited left ventricular systolic dysfunction (i.e., cardiomyopathy). Furthermore, among these patients, 16% experienced clinically evident heart failure NYHA class III–IV. Cardiomyopathy and overt heart failure occurred less frequently in the group that received anthracycline but not trastuzumab treatment, with respective incidence rates of 8% and 1% [6].

The HER2-signaling pathway plays a critical role in promoting the survival and adaptive responses of cardiomyocytes to various stressors. At the myocardial level, trastuzumab exerts its effects on many cell types that exhibit the expression of HER2 receptors, hence initiating pathogenic mechanisms. These include interstitial fibrosis, cellular dysfunction and apoptosis, microvascular injury, oxidative stress, and inflammation [7]. All of these processes are known to contribute to cardiac remodeling and the development of heart failure with reduced and preserved ejection fraction [8,9].

There is substantial evidence for both subclinical and overt left ventricular (LV) systolic dysfunction, which serve as defining points for trastuzumab-induced cardiotoxicity (TIC). Currently, there is insufficient information regarding right ventricular (RV) dysfunction, rhythm disturbances, and heart failure with preserved ejection fraction (HFpEF) in the context of trastuzumab therapy. Understanding the pathophysiology and all clinical manifestations of TIC is crucial for its early detection and cardioprotection administration. On the other hand, preventing cardiac damage will enable the successful completion of antitumor therapy.

## 2. Pathophysiology of Trastuzumab-Induced Cardiotoxicity

### 2.1. Trastuzumab’s Action on Tumor Cells

Trastuzumab is a recombinant monoclonal humanized antibody. It specifically targets the ErbB2 (v-erb-b2 avian erythroblastic leukemia viral oncogene homolog 2) receptor, also called HER-2. The ErbB2 receptor is a member of the human epidermal growth factor receptor family, which includes ErbB1, ErbB3, and ErbB4. These receptors regulate several cellular processes, such as metabolism, growth, division, differentiation, adhesion, function, and programmed cell death [10]. Tumor cells that overexpress ErbB2 exhibit uncontrolled proliferation due to ligand-independent heterodimerization of ErbB2 and ErbB3 and the activation of downstream intracellular phosphoinositide-3-kinase (PIK3) and Akt signaling pathways. Trastuzumab exerts its antitumor effects by disrupting ErbB2-ErbB3 heterodimers, inhibiting cell proliferation. ErbB2 receptor dephosphorylation and antibody-mediated immunological cytotoxicity are additional antitumor mechanisms [10,11].

### 2.2. NRG-1/ErbB Signaling Pathway’s Role in Heart Physiology

The unexpected discovery of trastuzumab-induced cardiac dysfunction provided a scientific impetus to elucidate its underlying mechanisms and the involvement of ErbB2 and other ErbB receptors in the physiological processes of the adult heart. Before identifying the deleterious cardiac effects of trastuzumab, it had already been demonstrated that ErbB signaling was involved in fetal heart morphogenesis. Experiments have demonstrated that the absence of the genes responsible for ErbB2, ErbB3, and ErbB4, or their ligand neuregulin-1 (NRG-1), disrupts the embryonic development of the heart, resulting in abnormal trabecularization of the heart chambers, impaired formation of the valvular apparatus, interatrial and interventricular septum, and, in severe cases, embryonic death [10]. NRG-1 also participates in the process of embryonic cardiomyocyte differentiation into conduction system cells [12].

The presence of ErbB1, ErbB2, and ErbB4 receptors and their corresponding ligands (EGF (epidermal growth factor) for ErbB1, NRG-1 for ErbB4, and HB-EGF (heparin-binding epidermal growth factor-like growth factor) for ErbB1 and ErbB4) persists beyond the neonatal period, suggesting their involvement in adult cardiac physiological processes. This was demonstrated by the development of dilated cardiomyopathy in postnatally mutated ErbB2 or ErbB4 mice [13,14]. NRG-1 is an EGF-like growth factor, a transmembrane protein released in the proximity of cardiomyocytes by endothelial cells of the endocardium or small vessels. The protein exhibits an affinity for ErbB3 in tumor cells and ErbB4 in cardiomyocytes. The subsequent interaction elicits structural modifications in these receptors, facilitating the assembly of ErbB3-ErbB3 and ErbB4-ErbB4 homodimers as well as ErbB2-ErbB3 and ErbB2-ErbB4 heterodimers. Within the cardiomyocytes, NRG-1 facilitates the generation of ErbB2/ErbB4 heterodimers and the subsequent activation of signaling pathways, including ERK1/2 and PI3-kinase/Akt. These cascades elicit a hypertrophic response and prevent apoptosis, respectively [9]. Again, through ErbB4, NRG-1 maintains mechanical and electrical connections among cardiomyocytes [12]. Furthermore, the NRG-1/ErbB system interacts with neurohormonal autoregulatory mechanisms. NRG-1 diminishes the inotropic cardiac response to adrenergic stimulation, while its deficiency leads to a decrease in the antiadrenergic effect exerted by the parasympathetic nervous system. On the other hand, the regulation of NRG-1 production and secretion is governed by the adrenergic nervous system and the renin–angiotensin–aldosterone system (RAAS) [12]. In summary, the actions of NRG-1 encompass the promotion of angiogenesis, cell elongation, cell adhesion, antiapoptotic pathways, hypertrophy, mitosis, and reduced susceptibility to adrenergic stimulation. All of these mechanisms are proposed to underlie the “cardioprotective” effect of the NRG-1/ErbB signaling pathway [10].

### 2.3. NRG-1/ErbB Signaling Pathway in Heart Failure Pathophysiology

The development of dilated cardiomyopathy in ErbB2-deficient mice and anti-HER2 (ErbB2)-targeted therapy patients suggests that the NRG-1/ErbB signaling pathway is implicated in the pathophysiology of heart failure [10]. The NRG-1/ErbB signaling pathway demonstrates a biphasic model of activity throughout the progression of chronic HF [8]. Following an initial compensatory activation, a phase of inhibition occurs, coinciding with the advanced stage of the disease [10]. Experiments with LV pressure overload revealed that NRG-1 was upregulated when concentric hypertrophy occurred but decreased when eccentric hypertrophy and systolic dysfunction developed, the latter likely due to increased sympathetic activation and angiotensin II and adrenaline release [10,12]. The decrease in endothelial NRG-1 production may result from the need for inotropic cardiac stimulation in the presence of pump failure [10,12]. Another investigation revealed that patients with chronic HF due to dilated cardiomyopathy or ischemic etiology had significantly elevated serum levels of the soluble fraction of ErbB2 released from the membrane upon activation. The levels rose as the patients’ functional class increased and their left ventricular ejection fraction (LVEF) decreased [15]. Other researchers offered additional evidence for the role of the ErbB signaling pathway in the development of peripartum cardiomyopathy and LV dilatation [16]. They support the notion that the ErbB signaling pathway plays a pathophysiological role in the onset of HF as it is involved in the adaptation of cardiomyocytes to stressors such as pregnancy, anthracycline exposure, ischemia, and hemodynamic load [16]. Furthermore, the functional status of ErbB/NRG-1 signaling may be related to the prognosis of CHF. According to a study by Ky et al. (2009), serum levels of NRG-1 predicted mortality and were significantly higher in HF patients with a poor prognosis [17].

### 2.4. Trastuzumab’s Effects on Cardiomyocytes

**Figure 1:**
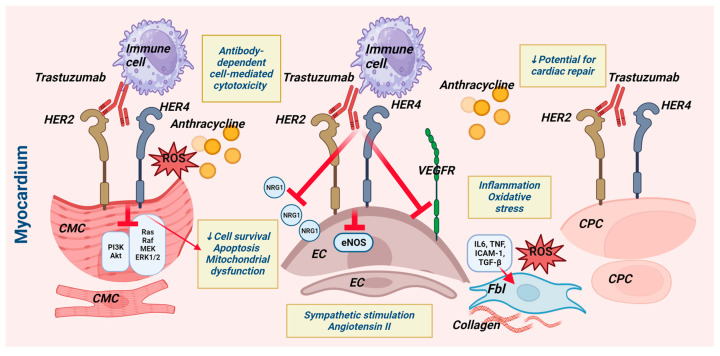
Pathophysiology of TIC. The adverse effects of trastuzumab within the myocardium involve interactions among various cell types that express HER2 receptors—cardiomyocytes, endothelial cells, and cardiac progenitor cells. By blocking the heterodimerization of HER2/HER4, trastuzumab inhibits downstream prosurvival pathways. In addition, immune cell activation, inflammation, reactive oxygen species generation, fibroblast stimulation, and collagen deposition in the extracellular matrix all play a role in myocardial injury. Furthermore, anthracycline exposure promotes these changes, making cardiomyocytes susceptible to damage and reliant on the NRG-1/HER2/HER4 pathway. Sympathetic and angiotensin II activation exert complementary roles in the evolution of cardiac damage. Akt—protein kinase B; CMC—cardiomyocyte; EC—endothelial cell; eNOS—endothelial nitric oxide synthase; ERK1/2—extracellular signal-regulated kinase 1/2; CPC—cardiac progenitor cell; Fbl—fibroblast; HER2—human epidermal growth factor receptor 2; HER4—human epidermal growth factor receptor 4; IL-1—interleukin-1; ICAM-1—intercellular adhesion molecule 1; MEK—mitogen-activated protein kinase; NO—nitric oxide; NRG1—neuregulin-1; PI3K—phosphoinositide 3-kinase; Raf—Raf kinase; Ras—Ras protein; ROS—reactive oxygen species; TGF-β—transforming growth factor-β; TNF—tumor necrosis factor; VEGFR—vascular endothelial growth factor receptor (created by Svetoslava Slavcheva using BioRender.com).

Trastuzumab can promote cardiomyocyte apoptosis by suppressing the NRG-1/ErbB2/ErbB4-Ras/Raf/MEK/ERK1/2 signaling pathway, which is crucial for cell survival (Figure 1). Trastuzumab blocks the NRG-1/ErbB2/ErbB4/PI3K/Akt pathway, inhibiting an inherent mitochondrial antiapoptotic pathway and leading to mitochondrial dysfunction [7]. In this manner, trastuzumab makes cardiomyocytes more susceptible to other detrimental and stressful factors, such as anthracycline. There is increased cardiomyocyte HER2 expression after anthracycline administration as an adaptive response to prevent cell death, which explains the mechanism of cardiotoxicity of the combined anthracycline/anti-HER2 therapy [18]. Angiotensin II, a key inhibitor of the NRG-1/ErbB2/ErbB4 signaling pathway and another inducer of apoptosis, is increased by trastuzumab when used with anthracyclines. Angiotensin II, on the other hand, stimulates NADPH oxidase in cardiomyocytes and other cells, increasing the production of oxygen radicals and, ultimately, leading to apoptosis. Trastuzumab’s synergistic effect with anthracyclines to suppress topoisomerase IIβ and thereby cause cell apoptosis is another molecular mechanism for the cardiotoxic action of the drug [7].

Therefore, the primary theory underlying TIC is that trastuzumab impairs the mechanisms required for cell survival after toxic exposure such as anthracycline therapy. The notion elucidates the rationale behind the observed escalation in cardiotoxicity incidence when anthracyclines and trastuzumab are co-administered [7]. Experimental studies conducted on human-induced pluripotent stem cell-derived cardiomyocytes (HPSC-CMs) provide evidence supporting the protective function of the NRG-1/ErbB2/ErbB4 signaling pathway in cardiomyocytes exposed to anthracyclines. One notable finding is that administering trastuzumab ± NRG-1 without anthracycline did not lead to cardiomyocyte injury. This fact raises the possibility of additional mechanisms underlying the cardiotoxicity of trastuzumab when used alone without prior anthracycline exposure [19].

In experimental studies on animals treated with trastuzumab with or without anthracyclines, apoptosis of cardiomyocytes, myocardial fibrosis, lymphocyte infiltration, and ultrastructural changes affecting mitochondria (edema, membrane rupture, and changes resembling oxidative stress) were observed [20,21,22,23]. Other animal studies demonstrated T-tubule disorganization and evidence of electrical instability (late afterdepolarizations, prolonged action potentials, and spontaneous calcium release from the sarcoplasmic reticulum) with anthracycline treatment but not trastuzumab treatment. However, these alterations were further intensified following the administration of trastuzumab [24]. Recent research on murine cardiomyocytes confirmed that mitochondrial dysfunction and oxidative stress are some of the mechanisms underlying trastuzumab’s adverse effects. In addition, it unveiled the occurrence of ferroptosis, a distinct type of iron-dependent cell death [25]. Some structural changes were observed in cardiac myofibers, such as decreased myofibrillar thickness and mitochondrial density, suggesting a potential reduction in contractile function. Genomic alterations responsible for contractile function, DNA repair, and mitochondrial function were discovered in murine cardiomyocytes exposed to trastuzumab [23]. According to some authors, when interpreting the animal-derived data, it should be considered that trastuzumab is a humanized antibody [10]. Nonetheless, the findings in animal models were replicated in human cardiomyocytes. Using cardiomyocyte cell lines derived from human pluripotent stem cells (HPSC-CMs), Necela, BM et al. (2017) demonstrated that trastuzumab inhibits the activity of ErbB2 and genes involved in calcium homeostasis, ischemic preconditioning, and the protective function of natriuretic peptides at the onset of atrial fibrillation and dilated cardiomyopathy in cardiomyocytes. Furthermore, the researchers observed modified gene expression related to glucose metabolism, decreasing glucose utilization. They also identified gene changes associated with mitochondrial function, namely, poorer membrane transport and reduced oxidative phosphorylation [26]. A study by Kitani T. et al. (2019) revealed that the contractility of HPSC-CMs was impaired during exposure to trastuzumab, similar to the observed effects of anthracyclines. Contrary to anthracycline-induced injury with sarcomere disorganization and increased troponin I release, these signs of cardiomyocyte damage were not observed in trastuzumab-exposed cell lines [27]. Moreover, there was an increase in the expression of genes implicated in the development of mitochondrial dysfunction, oxidative phosphorylation, ventricular dilatation, and dysfunction. The authors believe that mitochondria and impaired energy metabolism play a crucial role in the evolution of TIC. They also confirmed these functional alterations in cardiomyocytes derived from pluripotent stem cells of patients with already established trastuzumab-induced cardiomyopathy and reduced LVEF [27]. Mohan N. et al. (2016) found that trastuzumab suppressed autophagy in human cardiomyocytes in response to HER2-mediated Erk/mTOR/Ulk1 signaling pathway simulation [28]. In addition, they demonstrated that dysregulated autophagy is the cause of the increase in ROS production. Given that autophagy is a critical mechanism for maintaining cellular integrity and homeostasis, Mohan’s discoveries provide an additional rationale for the anthracycline–trastuzumab combination’s enhanced cardiac effects. After exposure to anthracycline, the ability of cardiomyocytes to perform self-repair is compromised, leading to oxidative stress and, eventually, apoptosis [28].

Thus, the findings suggest that trastuzumab works through the NRG-1/HER2 and angiotensin II/AT1 signaling pathways, as well as the downregulation of topoisomerase II activity. As a result, gene changes, mitochondrial dysfunction, autophagy dysregulation, and increased oxidative stress occur, making cardiomyocytes susceptible to other stressors such as anthracyclines and, ultimately, leading to apoptosis [7,27,28].

### 2.5. Effects of Trastuzumab on Other Heart Cell Types (Figure 1)

#### 2.5.1. The Influence on Cardiac Progenitor Cells

Cardiac progenitor cells (CPCs) are an endogenous subset of cells aiding cardiac repair [29]. Studies have shown that anthracyclines have adverse effects on CPCs, reducing the potential for myocardial repair [7]. Barth AS et al. (2012) discovered that CPCs also express ErbB2 at levels comparable to those expressed by mature cardiomyocytes. The scientists demonstrated that one of the potential mechanisms of trastuzumab’s cardiotoxicity is via its action on resident cardiac stem cells. Trastuzumab was found to impair the ability of CPCs to undergo cardiogenic differentiation, form a microvascular network, and promote myocardial recovery after infarction [30].

#### 2.5.2. Trastuzumab’s Effects on Endothelial Cells

Endothelial cells constitute the most abundant noncardiomyocyte cardiac cell population, and their functional deterioration has been linked to the onset of diverse cardiac pathologies [31,32]. In response to endothelial cell activation or damage, growth factors such as vascular endothelial growth factor (VEGF) are secreted, mobilizing endothelial progenitor cells for vascular repair [33]. Damaged endothelial cells also release inflammatory factors, like IL-6, TNF, and intercellular adhesion molecule 1 (ICAM1), triggering signal cascades that initiate inflammatory processes, producing reactive oxygen radicals and activating platelets [34]. A considerable body of research indicates that anthracycline exposure damages endothelial cells, a process which is mediated primarily by oxidative stress [7].

Trastuzumab has an antiangiogenic effect on tumors, as evidenced by a decrease in microvascular density and VEGF expression [34]. Endothelial cell injury’s contribution to TIC development is theoretically supported by the fact that endothelial cells contain ErbB2 receptors and are a source of the ligand NRG-1, which is required for ErbB2/ErbB4 signaling (Figure 1). The NRG-1/ErbB2/ErbB4 signaling pathway plays a role in stress-induced cardiac remodeling through paracrine interactions between endothelial cells, cardiomyocytes, fibroblasts, and macrophages, as well as via the autocrine regulation of endothelial function by inducing endothelial cell genomic reprogramming and an angiogenic response [35]. Trastuzumab reduces endothelial eNOS (endothelial NO synthetase) expression and NO production through NRG-1 action suppression, an increased production of reactive oxygen radicals, and angiotensin II [36]. These mechanisms are responsible for endothelial dysfunction and impaired vasodilator function, which can contribute to the onset of heart failure [7,36].

One study on mice addressed the impact of anthracyclines and trastuzumab on myocardial vasculature. The study revealed elevated subendothelial and interstitial collagen deposition, endothelial cell damage with increased vessel wall permeability, and protein extravasation [37]. The echocardiographic evaluation showed normal LVEF but elevated left atrial pressure and LV diastolic dysfunction. The research team hypothesized that these findings were consistent with the histological observation of progressive connective tissue deposition in the myocardium. The observed endothelial cell abnormalities explain diastolic cardiac dysfunction, which can lead to HFpEF [37]. Microvascular disorders are increasingly recognized as playing a significant role in the pathophysiology of HFpEF [33,38]. Wilkinson, EL et al. (2016) used human endothelial cell lines of dermal vascular, cardiac, and cerebral microvascular origin to examine the independent effect of trastuzumab in vitro [39]. They discovered that trastuzumab impaired endothelial barrier function, specifically disrupting the intercellular connections among endothelial cardiac microvascular cells. Moreover, it was noted that these effects exhibited greater prominence in the presence of anthracyclines, such as doxorubicin. Trastuzumab did not exhibit similar effects on the cerebral or cutaneous small vessels [39]. Coppola, C. et al. (2016) discovered an early onset of capillary rarefaction and cardiomyocyte apoptosis in trastuzumab-treated mouse hearts [40]. These changes coincided with early functional cardiac changes, as evidenced by a reduction in radial strain on echocardiography [40]. Gambardella et al. (2017) concluded that the participation of endothelial cells in intercellular communication (i.e., crosstalk) in the heart is relevant to the development of trastuzumab-induced cardiotoxicity [41]. Under normal conditions, cardiac endothelial cells release NRG-1 in response to oxidative stress, activating ErbB2/ErbB4 dimerization and the signaling pathways responsible for cardiomyocyte survival. ErbB2/ErbB4 signaling, on the other hand, stimulates NO production, which is essential for endothelial cell homeostasis [41].

#### 2.5.3. Trastuzumab’s Effects on Cardiac Fibroblasts

Trastuzumab promotes cardiac fibrosis, which is associated with the release of proinflammatory cytokines due to the production of free radicals by cardiomyocytes (Figure 1). Transforming growth factor-β (TGF-β) is one of these cytokines, and it stimulates fibroblast differentiation into myofibroblasts. Excessive extracellular matrix production impairs the electrophysiological properties of the myocardium and results in heart failure [7]. A study in mice showed that trastuzumab led to cardiomyocyte apoptosis, a reduction in capillary density, and the onset of fibrosis, which affected cardiac function with a decrease in LV fractional shortening [40].

#### 2.5.4. Trastuzumab and Cells of the Inflammatory Response

An essential mechanism of trastuzumab’s action as an antibody is the attraction of immune response cells to HER2-expressing cancer cells. Immune cells attack HER2-expressing cardiomyocytes and endothelial cells using this exact mechanism. Therefore, the cardiotoxic effects of trastuzumab are additionally facilitated through antibody-dependent cellular cytotoxicity [7]. Trastuzumab-related oxidative stress also induces cardiac inflammation. Experimental evidence with animals has demonstrated elevated levels of proinflammatory cytokines, namely, IL1, IL6, TNFα, TGF-β, and nuclear factor-kappa B (NF-κβ), along with superoxide compounds, serving as indicators of the presence of an inflammatory response. Mice and rabbits with ultrasound-confirmed trastuzumab-induced cardiotoxicity exhibited myocardial lymphocyte and macrophage infiltration [21,42].

### 2.6. Mechanisms of Trastuzumab-Induced Cardiotoxicity: A Summary

Trastuzumab exhibits cytotoxic effects on cardiomyocytes and diverse myocardial cell populations (Figure 1). Pathological processes facilitated by intercellular communication among multiple cell types, including cardiomyocytes, endothelial cells, fibroblasts, and inflammatory cells, result in cardiac remodeling. Microvascular injury, interstitial fibrosis, an inflammatory response, and the dysfunction and apoptosis of cardiomyocytes are among the effects of trastuzumab. Consequently, both diastolic and systolic myocardial dysfunction may develop, eventually resulting in heart failure (Figure 1, Graphical Abstract).

In 2005, Ewer et al. stated that anthracycline-induced and trastuzumab-induced cardiomyopathies’ mechanisms and clinical presentations are fundamentally distinct [43]. They noted that TIC is not dose-dependent, is generally fully reversible, and does not demonstrate structural or ultrastructural changes in the myocardium, despite the researchers relying on a limited number of histological studies. Anthracyclines, on the other hand, cause structural, irreversible changes in the myocardium with increasing cumulative doses. The authors proposed categorizing cardiac dysfunction caused by antitumor drugs into type I with the prototype anthracycline therapy and type II with the prototype trastuzumab therapy [43]. This concept has been questioned because of the scientific evidence that has accumulated over time. An ongoing debate exists regarding classifying myocardial damage induced by antitumor therapy [44,45].

One counterargument to the classification proposed by Ewer et al. involves data indicating irreversible cardiac injury associated with trastuzumab treatment. Additionally, a higher occurrence of cardiac dysfunction is observed in patient cohorts who received trastuzumab therapy without anthracyclines compared to cohorts treated with anthracycline alone [44,45]. It is believed that the long-term cardiovascular effects of trastuzumab administration are underestimated [45]. The categorization of type II cardiotoxicity as a distinct entity with more favorable outcomes based on the potential for myocardial damage reversibility is considered to be incorrect [45]. The claims presented are substantiated by a population-based study conducted by Goldhar et al. (2015), encompassing a cohort of more than 19,000 individuals diagnosed with breast carcinoma. The incidence of cardiac dysfunction was 2.5% in the anthracycline-only subpopulation and 5.1% in patients treated with trastuzumab without anthracyclines [46]. Trastuzumab therapy was estimated to be an independent factor that increases the risk of developing heart failure by sixfold (HR = 5.77, *p* = 0.001) [47]. Chen et al. (2012) found comparable results in their population-based investigation of over 45,000 people aged 66 and older [47]. The cumulative incidence of heart failure increased in the years following anti-HER2 administration, indicating that cardiac dysfunction was irreversible. Moreover, the cumulative incidence with trastuzumab-only treatment was considerably higher: 15.7%, 20.7%, and 26.7% for 1, 2, and 3 years, respectively, compared to 7.8%, 12.4%, and 17% with anthracycline treatment alone [47].

The proponents of the theory of Ewer et al. (2005) asserted that anthracycline-induced and trastuzumab-induced cardiac deterioration differ in their mechanisms of impairment and should therefore be distinguished [44]. Against the evidence of irreversible trastuzumab-induced myocardial injury and troponin I release, they propose the concept of a vulnerable cardiomyocyte pool after anthracycline administration. Depending on the severity of the initial impact, the subsequent blocking of ErbB2 signaling and the deprivation of the cell’s ability to repair can lead to irreversible cardiomyocyte damage [44]. In other words, cases involving irreversible myocardial dysfunction and necrosis do not contradict the type I and type II cardiotoxicity classifications. Another rationale for their classification is that trastuzumab has been used for multiple years in metastatic cancer patients without showing any signs of cardiotoxicity. There have also been instances in which the drug was successfully re-administered after developing cardiac dysfunction, giving it a better long-term profile [44,48].

However, the current guidelines of the European Society of Cardiology (ESC), which were developed in collaboration with the International Cardio-Oncology Society (IC-OS), do not differentiate between type I and type II cardiomyopathy [49].

#### What Evidence Exists Regarding Structural Myocardial Damage Caused by Trastuzumab?

There is little direct evidence of the histological and ultrastructural changes in the myocardium induced by trastuzumab. Few studies have employed endomyocardial biopsies. In their seminal study, Ewer et al. (2005) performed endomyocardial biopsies on nine patients with myocardial dysfunction receiving sequential trastuzumab therapy (after anthracyclines) [43]. They did not find structural and ultrastructural changes similar to those observed in anthracycline-induced cardiotoxicity [43]. Indirect evidence of structural alterations in the myocardium is provided by cardiac magnetic resonance imaging (MRI) studies demonstrating subepicardial linear postcontrast enhancement of the lateral LV wall in a small population with echocardiographic or MUGA-proven trastuzumab-induced cardiac dysfunction [50,51]. In a small number of patients (n = 3) with subclinical cardiac dysfunction associated with trastuzumab, Thavendiranathan et al. observed areas of focal myocardial edema (prolonged T2 relaxation time), as well as areas of myocardial fibrosis—late gadolinium enhancement (LGE) [52]. Another study questions the results of the previously mentioned research by observing that LGE, predominantly of the ischemic type with subendocardial or transmural involvement of the myocardium, was detected in only 10% of patients who received anthracyclines with or without trastuzumab treatment [53]. In the majority of the remaining cases of nonischemic LGE, alternative causes were identified, such as myocarditis (viral and related to immune checkpoint inhibitors), sarcoidosis, acute myocardial calcification, and lymphoma infiltration. The study challenges the evidentiary value of the MRI technique for the unambiguous interpretation of structural changes caused by trastuzumab’s cardiotoxicity [53].

## 3. Trastuzumab-Induced Cardiotoxicity from a Clinical Perspective

Asymptomatic left ventricular systolic dysfunction with decreased ejection fraction is the most common manifestation of cardiotoxicity caused by trastuzumab. The prevalence of heart failure with a reduced ejection fraction (HFrEF) is comparatively lower. Following the disclosure of these conditions by Denis Slamon, in 2001, a committee known as the Cardiac Review and Evaluation Committee (CREC) was formed [54]. The committee proceeded to establish a set of criteria for the diagnosis of cardiac dysfunction: (1) cardiomyopathy characterized by a decrease in the left ventricular ejection fraction (LVEF), global or more pronounced for the septum; (2) symptoms of congestive heart failure; (3) signs associated with congestive heart failure, such as T3 gallop, tachycardia, or both, and others; (4) a decrease in the LVEF of at least 5% to less than 55% with concomitant signs and symptoms of congestive heart failure or a decrease in the LVEF of at least 10% to less than 55% without accompanying signs and symptoms of heart failure. Each of the above four criteria is deemed adequate to establish a diagnosis of cardiac dysfunction [54].

### 3.1. Heart Failure and Left Ventricular Systolic Dysfunction: Large Randomized Controlled Trials’ Data

The subsequent randomized trials, namely, NSABP B-31, NCCTG N9831, HERA, and BCIRG-006, implemented more stringent patient selection criteria [55,56,57,58] (Table 1). They excluded individuals with elevated cardiovascular risk due to uncontrolled hypertension, arrhythmias, valvular heart disease, coronary artery disease, heart failure, or asymptomatic left ventricular systolic dysfunction. Rigorous protocols were implemented to monitor cardiac function. Chemotherapy regimens were modified by employing sequential anthracycline and trastuzumab protocols rather than concurrent administration, and only after establishing a normal postanthracycline LVEF [55]. As a result, the incidence of cardiotoxic events was lower than Denis Slamon initially reported in 2001. In both trials, NSABP B-31 and NCCTG N9831, the incidence of heart failure and cardiovascular mortality was 2.0% among patients receiving trastuzumab therapy compared to a rate of 0.45% in the chemotherapy-alone group. A significant proportion of patients, 86.1%, exhibited complete or partial recovery from cardiac dysfunction [59]. However, upon considering the CREC criteria and asymptomatic cardiac dysfunction, the incidence of TIC in the NSABP B-31 trial was considerably greater. Specifically, the rate was 34% (95% CI: 31% to 38%) in the trastuzumab arm and 17% (95% CI: 15% to 20%) in the group that did not receive trastuzumab (*p* = 0.0001). A significant proportion of patients in the trastuzumab group, 19%, discontinued treatment due to either asymptomatic LV systolic dysfunction or overt heart failure [55]. The NCCTG N9831 trial observed a 7.8–10.4% incidence of asymptomatic cardiomyopathy among trastuzumab-treated patients. In comparison, patients on nontrastuzumab regimens had a 4.0–5.1% incidence [56]. Only half of the patients restored their LV function and were able to resume their oncological medication. The HERA study collected data on cardiovascular events related to a two-year trastuzumab therapy regimen [57]. The two-year application of trastuzumab nearly doubled the incidence of cardiac events compared to a one-year application (9.4% in the two-year arm versus 5.2% in the one-year arm). The incidences of the asymptomatic LVEF decrease were 7.2% and 4.1%, respectively. After approximately 7.2 months, 87.2% of patients demonstrated complete LV function recovery, but one-third experienced an additional decline in LVEF to below 50% [57]. In the BCIRG-006 study, it was observed that 33% of the total 194 patients who were administered AC-T (doxorubicin and cyclophosphamide followed by docetaxel every 3 weeks) in combination with trastuzumab experienced a persistent reduction in left ventricular ejection fraction (LVEF) above 10% over a duration of four years [58]. One meta-analysis summarized data from 10 randomized controlled trials, including the aforementioned large studies [60]. The findings of this meta-analysis revealed that the incidence of heart failure was 1.7% when trastuzumab was applied sequentially to anthracyclines and 1.5% when it was given following nonanthracycline therapy. The percentage of patients with a decrease in LVEF was 7.5% and 5.2%, respectively. Trastuzumab increased the risk of developing LV systolic dysfunction by twofold (RR = 2.13, 95% CI, 1.31–3.49; *p* = 0.003) and of developing heart failure fourfold (RR = 4.19, 95% CI, 2.73–6.42; *p* < 0.00001) [60].

### 3.2. Heart Failure and Left Ventricular Systolic Dysfunction: Evidence from Real-World Clinical and Registry-Based Studies

However, actual clinical practice data differ from the results of randomized controlled trials (Table 1). A high frequency of heart failure or cardiomyopathy was discovered in registry-based studies of older populations with a mean age over 70. According to Chen et al. (2012), the incidence of new-onset heart failure increased in the years following cancer therapy, reaching 32.1% in the third year after combined trastuzumab and nonanthracycline-based therapy and up to 41.2% with anthracycline-based regimens [47]. The findings of the Chavez-MacGregor’s (2013) research team are comparable. Heart failure was detected in 29% of the trastuzumab-treated population, whereas anthracyclines were administered to only 60% of the patients [61]. Regarding younger patients, Thavendiranathan P. et al. (2016) reported that heart failure or cardiovascular death occurred in 5.1% to 6.6% of trastuzumab-treated patients with a mean age of 54 years [62]. Another research group examined a population of a similar age to that of Thavendiranathan et al. (2016). Heart failure or cardiovascular mortality after treatment with trastuzumab was found to occur at a higher rate in the fifth year compared to the first, ranging from 12.1% to 20% [63]. Trastuzumab increased the risk of cardiac events fourfold if given without anthracyclines and sevenfold when given sequentially after anthracycline-based chemotherapy [63]. Guglin et al. (2009) reported that one-third of 156 trastuzumab-treated patients with a mean age of 51.4 years had cardiomyopathy, defined as heart failure or a drop in LVEF of at least 10% or less than 50%, which resulted in the permanent cessation of medication in 10% of patients with TIC [64]. One-third of the population studied by Guglin et al. (2009) had at least one cardiovascular comorbidity [60]. Data on patients (n = 499) from 10 Italian oncological centers led Tarantini et al. (2012) to conclude that TIC is a frequent event, being in most cases asymptomatic with a reversible reduction in LVEF. LV systolic function declined in the first three months after trastuzumab initiation [65]. In contrast to randomized studies, the mentioned studies report cardiotoxic events in actual clinical practice, and the patients in the studied populations have a higher cardiovascular risk.

Current ESC cardio-oncology guidelines identify asymptomatic cancer-therapy-related cardiac dysfunction (CTRCD) based on systolic LV function estimation [49]. An LVGLS decline of 15% or greater, which may be corroborated by an increase in cardiac troponins or BNP, identifies the mildest form. The most severe forms are characterized by a decrease in LVEF below 40%. A moderate form of asymptomatic CTRCD should be acknowledged when the LVEF drops by ≥10 percentage points to an LVEF of 40–49% or <10 percentage points to an LVEF of 40–49%, together with an LVGLS decline of >15% or a new increase in cardiac biomarkers [49].

### 3.3. Trastuzumab and Rhythmic Abnormalities

Generally, TIC research has focused on mechanical cardiac function. There is a paucity of data regarding rhythm and conduction disturbances, and it remains uncertain whether they can manifest independently or are causally linked to the onset of cardiac dysfunction. A review by Siri-Angul et al. summarizes the current data on electrophysiological disturbances associated with trastuzumab treatment [66]. There have been reports of nonspecific arrhythmias, sinus bradycardia, clinical cases involving ventricular arrhythmias, sudden cardiac death, and left and right bundle branch blocks. The authors assert that there is a need for research that particularly addresses rhythm pathology as a potential consequence of HER2 therapy [66].

### 3.4. Right Ventricular Dysfunction

Although recommendations for the surveillance of patients receiving potentially cardiotoxic therapy include the assessment of right ventricular (RV) function, its follow-up is rarely reported. RV dysfunction is not a starting point for defining cardiotoxic events. After sequential therapy with anthracyclines and trastuzumab, Santoro C. et al. (2021) discovered that a significant proportion (66%) of patients experienced decreased longitudinal RV function measured via speckle tracking [67]. No statistically significant alterations were observed in the standard echocardiographic parameters of RV function. The longitudinal strain of the left ventricle also decreased significantly, although to a lesser degree [67]. In a retrospective study, Calleja et al. (2015) discovered that the patients (n = 30) with proven TIC had poorer RV function than the control group. The researchers used the RV parameters fractional area change (RVFAC) and global longitudinal strain (RVGLS). In 40% of the patients with previously documented LV systolic failure, the RVGLS dropped below 20% [68]. Bendahou et al. (2023) found biventricular dysfunction in 16.8% of patients with TIC defined by TAPSE < 17 and S′< 9.5, and only in 50% of these patients was the RV dysfunction reversible [69]. Grover et al. (2013) employed magnetic resonance imaging (MRI) to analyze the left and right ventricular parameters of 46 women receiving anthracycline, trastuzumab, or both [70]. The right ventricular ejection fraction (RVEF) decreased by 10% in 23% of the population after 4 months of treatment and in 34% of the patients after 12 months. The RVEF was below the lower normal limit of 50% in eight patients. However, their research did not distinguish between the effects of trastuzumab and anthracyclines [70]. Similar findings were reported by Barthur et al. (2017) in a prospectively examined group (n = 41) which underwent trastuzumab therapy and MRI monitoring [71]. They reported a minor but significant decrease in RVEF during the therapy, with 23% of the population having an RVEF less than 51% at six months [71]. One clinical case described isolated right ventricular failure following trastuzumab treatment [72]. Using the echocardiographic indicators TAPSE, RV TDI MPI, and E/e′, Kılıçaslan et al. (2015) demonstrated RV systolic and diastolic dysfunction six months following trastuzumab therapy initiation [73]. A meta-analysis of 15 studies involving 644 patients revealed that antitumor treatment with trastuzumab with or without anthracyclines led to a statistically significant decline in RV function, as measured using the ejection fraction, fractional area change, and longitudinal RV free wall strain [74]. According to this meta-analysis, the parameters RVGLS and TAPSE did not change significantly [74].

### 3.5. Heart Failure with Preserved Ejection Fraction

Concerning the development of HFpEF, scientific studies have focused on echocardiographic parameters that determine the presence of diastolic dysfunction and elevated left atrial pressure. According to the 2021 ESC recommendations, the definition of HFpEF includes the presence of symptoms, objective evidence of cardiac structural, and/or functional abnormalities consistent with LV diastolic dysfunction or increased LV filling pressures, as well as elevated natriuretic peptide levels [75]. Hence, the finding of abnormal LV diastolic performance alone is insufficient to establish the presence of heart failure.

#### Diastolic Dysfunction

LV diastolic dysfunction precedes LV systolic deterioration in cardiovascular diseases like ischemic heart disease and arterial hypertension [76,77]. Several research teams investigated the progression of diastolic dysfunction as a manifestation of the cardiotoxic effects of antitumor therapy and the temporal relationship between its onset and the impairment of LV systolic function. The hypothesis that the cardiotoxic effects of antitumor therapy may lead to an earlier onset of diastolic dysfunction may facilitate more timely identification of the cardiac damage caused by oncological treatment and, consequently, earlier initiation of preventative treatment.

The development of diastolic dysfunction during breast cancer therapy was addressed in a limited prospective study using echocardiographic criteria: E/e′ and E/A [78]. Patients were divided into three groups based on adjuvant therapy: trastuzumab and left-sided radiotherapy (n = 40), trastuzumab and right-sided radiotherapy (n = 32), and radiotherapy alone (n = 71). Within six months, 32% to 37% of patients in the targeted treatment groups developed diastolic dysfunction, compared to only 19% in the radiotherapy-alone group [74]. Simultaneously, the percentage decrease in LVEF was modest in each of the three groups: 2.5%, 3%, and 0%, respectively. Trastuzumab therapy was found to be one independent predictor (HR 1.35, *p* = 0.025) of the development of diastolic dysfunction after accounting for the impact of comorbidity, age, BMI, anthracycline- and taxane-chemotherapy. It is essential to acknowledge that adjuvant therapy with anthracyclines was administered to approximately 80% of the population [78]. In a retrospective study of 129 patients treated with trastuzumab, 13% of patients (n = 17) exhibited LV diastolic dysfunction as measured by E/e′> 15, with only 7% exhibiting a decrease in LVEF [79]. The LVEF decline occurred concurrently or after the onset of the diastolic impairment [79]. Another retrospective observational study using a MUGA scanner investigated the time dependency of diastolic and systolic LV dysfunction onset in patients with breast cancer treated with trastuzumab and adjuvant chemotherapy [80]. The authors discovered that in most cases, LV diastolic failure preceded systolic dysfunction. One of the disadvantages of the study is its reliance on a maximum of four MUGA scans conducted during a timeframe of four to six months, potentially resulting in a delayed identification of diastolic dysfunction. On the other hand, it should be noted that more than 80% of patients received anthracycline chemotherapy and more than 80% received radiotherapy [80]. Hence, a conclusive determination cannot be made regarding the causative factor of diastolic dysfunction, whether it be chemotherapy, radiotherapy, or targeted therapy. The largest prospective study to date that monitored LV diastolic parameters in 360 patients divided them into three groups based on the type of antitumor therapy—doxorubicin, doxorubicin, and trastuzumab—and a third group receiving trastuzumab alone [81]. The study proved the early onset of LV diastolic dysfunction before the development of systolic failure in a high percentage of the studied population—70% in the second year. The researchers found decreases in the E/A ratio, and the e’ tissue velocities of the lateral and septal mitral annulus and an increase in E/e′ in patients treated with anthracyclines with or without trastuzumab. Interestingly, diastolic dysfunction was not observed in the trastuzumab-alone group [81]. Scientific data are scarce regarding clinically evident HFpEF. A 5-year retrospective observational study of an Asian population (n = 386) with breast cancer treated with trastuzumab revealed the incidence of major cardiovascular events and heart failure hospitalizations [82]. This study stratified cases of new-onset HF based on their documented ejection fraction. Overall, the incidence of hospitalizations for HF was 2.8% (n = 11/386), and the incidence of hospitalizations for HFpEF was approximately one-third of them (n = 4) [82]. It is important to note that the examined population had relatively low comorbidity, an average age of approximately 54 years, and few cardiovascular risk factors [82]. Using data from Taiwan’s national registry, the same research team compared two matched groups of over 12,000 breast cancer patients treated with and without trastuzumab. The hospitalization rate for HF (with reduced and preserved EF) was 3.21% in the trastuzumab group and 1.9% in the control group. The risk for heart failure was 62% greater in the trastuzumab therapy group (1623, 95% CI: 1305–2018, *p* < 0.001) [82]. Another case–control study assessed cardiorespiratory fitness (peak VO2) in breast cancer patients seven years following systemic therapy [83]. The authors compared the women by separating them into three groups: the first one with documented TIC during the therapy (asymptomatic decrease in LVEF) (n = 22), the second group without evidence of cardiotoxicity (n = 20), and the third, healthy controls (n = 15). Women with trastuzumab-induced symptomatic heart failure were excluded. Reasonably, the mean LVEF and GLS were significantly lower in the group with cardiotoxicity (LVEF 56.9% ± 5.2%; GLS −17.8% ± 2.2) than in the second and third groups (LVEF 62.4% ±4.0% and 65.3% ± 2.8%; GLS 9.8% ± 2.2% and −21.3% ± 1.8) [83]. Notably, 90% of the population was treated with anthracyclines. The groups were comparable in terms of cardiovascular comorbidities and risk factors. Mean peak VO2 (22.9 ± 4.4 mL/kg/min) was 15% lower in the group with prior cardiotoxicity compared to the group without prior cardiotoxicity (27.0 ± 5.3 mL/kg/min; *p* = 0.03) and 25% lower compared to healthy controls (30.5 ± 3.4 mL/kg/min; *p* < 0.001) [83]. In this study, researchers demonstrated that patients treated with trastuzumab experienced a long-term decline in physical capacity. Moreover, they indicated that impairments in LV systolic function after trastuzumab therapy are not transient but persist over time. Disturbances in longitudinal LV function appeared to predict reduced cardiorespiratory fitness (β coefficient, −0.75; 95% CI, −1.32 to −0.18) [83].

## 4. Conclusions and Future Directions

Trastuzumab exerts unfavorable short- and long-term effects on heart function, presenting as HF with reduced or preserved EF, asymptomatic LV systolic and diastolic dysfunction, and RV dysfunction. Understanding the manifestations of TIC enables its early detection and the implementation of cardioprotective therapy, thereby facilitating the successful completion of oncological treatment and the prevention of long-term consequences. Global longitudinal strain is a validated early marker of myocardial function impairment, also in the setting of trastuzumab-induced myocardial damage. The study conducted by Van der Linde et al. (2023) demonstrates that LVGLS change occurs prior to the decline in LVEF by approximately one month [84]. The European Society of Cardiology recommends considering starting neurohormonal cardioprotection after substantial reduction in GLS during ongoing maintained trastuzumab therapy [49]. However, there is still no evidence for the benefits of implementing cardioprotective treatment in cases of GLS decline in the setting of trastuzumab treatment. The three-year SUCCOUR trial in the anthracycline scenario did not yield superior outcomes in terms of final LVEF when comparing the use of cardioprotection (ACE inhibitor, ARB, and beta-blockers) after detecting GLS decline versus LVEF reduction [85]. Further research is required to obtain data on LV diastolic and RV dysfunction and to determine whether their detection should be a reason for introducing cardioprotective medications. The discovery of efficient cardioprotective drugs is dependent on understanding the pathophysiological pathways of TIC. Given the new arsenal of SGLT2 inhibitors available to cardiologists, these questions appear even more intriguing. SGLT2 inhibitors have demonstrated notable therapeutic advantages in the context of HF with a reduced and preserved ejection fraction due to their ability to activate autophagy and restore mitochondrial and cellular function [86]. Experimental data indicate the capacity of the SGLT2 inhibitor empagliflozin to counteract TIC, along with the associated oxidative stress, mitochondrial dysfunction, DNA damage, and apoptosis [87].

## Figures and Tables

**Table 1:** Summary of randomized controlled and observational real-world trials on manifestations of TIC.

STUDY	GROUPS	HF III–IV NYHA CLASS	HF	ASYMPTOMATIC DECREASE IN LVEF
**RANDOMIZED CONTROLLED TRIALS**				
**NSABP B-31** **(Tan-Chiu E, 2005) [55]**	(1) AC (4 cycles) + paclitaxel and trastuzumab (2) Paclitaxel without trastuzumab	**HF III–IV NYHA class + CV death** (1) 4.1% (n = 31/850) (2) 0.8% (n = 5/850)		**CREC criteria decreased LVEF** (1) 34% (2) 17%
**NCCTG N9831** **(Perez EA, 2008) [56]**	(1) AC + paclitaxel (2) AC + paclitaxel and trastuzumab (52 wks.) (3) Paclitaxel + trastuzumab (52 wks.)	**HF III–IV NYHA class + CV death** (1) 0.3% (2) 2.8% (3) 3.3%		**CREC criteria decreased LVEF** (1) 5.1% (2) 7.8% (3) 10.4%
**BIG 1-01—Extended 8-year HERA study Early-stage BC ** **(Evandro de Azambuja, 2014) [57]**	(1) Trastuzumab for 2 years (n = 1673) (2) Trastuzumab for 1 year (n = 1682) (3) No trastuzumab (n = 1744) 94.1% of patients are pretreated with anthracyclines	(1) 0.8% (2) 0.8% (3) 0.0%		**Decreased LVEF** (1) 7.2% (2) 4.1% (3) 0.9%
**BCIRG-006—Early-stage BC** **n = 3222** **(Slamon D, 2011) [58]**	(1) AC-T (2) AC-T + trastuzumab (52 wks.) (3) TCH		(1) 0.7% (2) 2% (3) 0.4%	**Decreased LVEF** (1) 11.2% (2) 18,6% (3) 9.4%
**Meta-analysis—10 RCTs on BC ** **(Chen T, 2011) [60]**	(1) Trastuzumab sequentially to anthracyclines (2) Trastuzumab concurrently with anthracyclines (3) Trastuzumab—nonanthracycline therapy		(1) 1.7% (2) 13.9% (3) 1.5%	**Decreased LVEF** (1) 7.5% (2) 30.4% (3) 5.2%
**OBSERVATIONAL REAL-WORLD AND REGISTER-BASED TRIALS**				
**(Chen J, 2012) [47] n = 45 537, mean age 76.2 (67 to 94) years**	(1) Trastuzumab ± nonanthracycline therapy (2) Anthracyclines + trastuzumab (3) Anthracyclines ± other chemotherapy (without trastuzumab) (4) Nonanthracycline therapy (5) No adjuvant therapy	**HF + cardiomyopathy** **1st and 3rd years** (1) 16.7% and 32.1% (2) 22% and 41.9% (3) 9.8% and 20.2% (4) 8.4% and 19.2% (5) 7.0% and 18.1%		
**(Chavez-MacGregor M, 2013) [61] n = 9525, mean age 71 years**	(1) Trastuzumab (2) Without trastuzumab		**HF** (1) 29.6% (2) 18.9%	
**(Thavendiranathan P, 2016) [62] n = 18,540, mean age 54 years N = 18,540, follow-up 3 years**	(1) Anthracyclines (without trastuzumab) (2) Trastuzumab + nonanthracycline-based therapy (3) Anthracyclines + trastuzumab (sequentially) (4) Another therapy (nonanthracycline, nontrastuzumab)		**HF + CV death** (1) 2% (2) 5.1% (3) 6.6% (4) 3.2%	
**(Bowles EJ & Team, 2012) [63] n = 12,500, mean age 60 years**	(1) Anthracyclines (without trastuzumab) (2) Trastuzumab (without anthracyclines) (3) Anthracyclines + trastuzumab (sequentially) (4) Another chemotherapy (5) Without chemotherapy		**HF + Cardiomyopathy** **1st and 5th years** (1) 1.2% and 4.3% (2) **3.6% and 12.1%** (3) **6.2% and 20.1%** (4) 1.3% and 4.3% (5) 0.9% and 3.1%	
**(Guglin M, 2009) [64] n = 118, mean age 51.2 years**	Trastuzumab 93% of patients—anthracycline-based therapy		**HFrEF + HFpEF** 11 (9.3%)	➢ 14.4%—LVEF < 50% ➢ 16.1%—**↓**LVEF ≥ 10%
**(Tarantini L, 2012) [65] n = 499, mean age 55 ± 11 years**	Trastuzumab 87% of patients—anthracycline-based therapy		**HF II NYHA class** 3%	➢ 20%—**↓**LVEF > 10% < 20% ➢ 3%—**↓**LVEF > 20% or LVEF < 50%

AC—doxorubicin and cyclophosphamide every 3 weeks; AC-T—doxorubicin and cyclophosphamide followed by docetaxel every 3 weeks; CREC—Cardiac Review and Evaluation Committee; CV—cardiovascular; HF—heart failure; HFpEF—heart failure with preserved ejection fraction; HFrEF—heart failure with reduced ejection fraction; LVEF—left ventricular ejection fraction; TCH—docetaxel and carboplatin plus 52 weeks of trastuzumab.

## Data Availability

Not applicable.
